# Examining HPO by organ and system to facilitate practical use by clinicians

**DOI:** 10.1186/s44342-024-00024-1

**Published:** 2024-11-12

**Authors:** Eisuke Dohi, Terue Takatsuki, Yuka Tateisi, Toyofumi Fujiwara, Yasunori Yamamoto

**Affiliations:** 1 National Center of Neurology and Psychiatry, National Institute of Neuroscience, Kodaira, Tokyo, Japan; 2 Database Center for Life Science, ROIS-DS, Kashiwa, Chiba Japan; 3 Office of NBDC Program, JST, Tokyo, Japan

**Keywords:** Large Language Model, Biological ontologies, Annotation, Phenotype

## Abstract

The Human Phenotype Ontology (HPO) is widely used for annotating clinical text data, and sufficient annotation is crucial for the effective utilization of clinical texts. It was known that the use of LLMs can successfully extract symptoms and findings, but cannot annotate them with the HPO. We hypothesized that one of the potential issue for this is the lack of appropriate terms in the HPO. Therefore, during the Biomedical Linked Annotation Hackathon 8 (BLAH8), we attempted the following two tasks in order to grasp the overall picture of HPO. (1) Extract all HPO terms for each of the 23 HPO subclasses (defined as categories) directly under the HPO "Phenotypic abnormality" and then (2) search for major attributes in each of 23 categories. We employed LLM for these two tasks related to examining HPO and, at the same time, found that LLM didn't work well without ingenuity for tasks that lacked sentences and context. A manual search for terms within each category revealed that the HPO contains a mix of terms with four major attributes: (1) Disease Name, (2) Condition, (3) Test Data, and (4) Symptoms and Findings. Manual curation showed that the ratio of symptoms and findings varied from 0 to 93.1% across categories. For clinicians, who are end-users of medical terminology including HPO, it is difficult to understand ontologies. However, for good quality ontology is also important for good-quality data, and a clinician’s help is essential. It is also important to make the overall picture and limitations of ontologies easy to understand in order to bring out the explanatory power of LLMs and artificial intelligence.

## Introduction

The Human Phenotype Ontology (HPO) [1], developed by the Jackson Laboratory, serves as a widely utilized tool for annotating phenotypes in clinical text data. The application of HPO in annotation enhances comprehensive searches for phenotypic information and facilitates mutual comparisons. Several attempts have been made to annotate symptoms, signs, and findings in clinical text. For example, in the SympTEMIS task in BioCreative VIII, using the text annotated by clinicians as the gold standard: (1) entity recognition, (2) entity normalization and linking, and (3) multilingualization were performed, and many teams worked with models using transformers [2]. PhenoTagger is a hybrid of two methods; a dictionary-based method for matching dictionaries based on HPO, and a deep learning model in the medical domain (BioBERT) for concept recognition and recognition of symptoms for paths not found in the dictionary. BioBERT is accurate, but it has been reported that limitations in finding unknown concepts and distinguishing between different concepts with the same name [3].

We have previously pointed out the importance of annotating detailed patient information in order to visualize patients with atypical symptoms in an easily understood manner [4]. Since LLM is developing rapidly, we decided to apply LLM hypothesized that it would be easier and more accurate to annotate clinical text using LLM and the visualization of patient information could be smoothly automated. In our previous trials, we found that LLM could extract symptoms, findings, and signs from clinical texts at a level sufficient for clinicians to evaluate. However, annotation by HPO was difficult, both for direct annotation to text and for annotation to the terms after extraction. There were two possible reasons for this: the annotation functionality may not be sufficient, and the HPO may not have appropriate terms.

To address the second problem mentioned above here, it is necessary to evaluate medical terminology, and the gold standard for this evaluation requires the cooperation of medical professionals, the end users who actually use and record the terms in the field. It is not difficult for medical professionals to understand individual terms, but it is difficult for them to grasp the whole picture of an ontology with a tree structure, and this is no exception in HPO [5]. Therefore, in BLAH8, we decided to try to visualize what kind of words and phrases exist in the HPO for each organ or system to make it easier for clinical specialists in each department to understand.

## Methods

### Extraction of lists of HPO terms in each category with LLM or TogoDX

Using ChatGPT(GPT-4 turbo), we attempted to extract terms in each category of HPO phenotypic abnormalities. We created GPTs to deal with the terms in HPO and uploaded 2 Excel files of HPO; “Definition.xlsx” contains “HPO id”, “Label”, “Definition” and “Synonyms” (available at GitHub) and “Parent_Child.xlsx” contains “HPO id”, “Label”, “Parent” and “Child” (available at GitHub).

The instructions of GTPs were as follows, “HPO Analyzer is designed to handle and analyze Human Phenotype Ontology (HPO) lists, focusing on extracting terms within specific categories and determining their hierarchical levels. The GPT performs comprehensive searches based on user instructions and outputs the results in Excel format. It prioritizes data accuracy and completeness, evaluating the reliability of extracted information and providing feedback to the user. The GPT responds with a technical, precise tone, while also being user-friendly and clear in explanations.”. The “Conversation starters” were as follows: “Extract all child terms under the category X.”,” Show the hierarchical level of terms within the category Y.”,” Provide an Excel sheet of terms under category Z.” and “Assess the reliability of extracted HPO data.”.

The following prompts were used to extract terms from HPO.

“Please create a csv file of the list of all terms included in the "category name (such as cellular phenotypic abnormalities)" of the phenotypic abnormalities of HPO, using the uploaded file and website as a reference”. All the outputs were evaluated from clinician’s perspectives.

We also employed TogoDX to obtain a list of terms in each category of HPO phenotypic abnormality. TogoDX is a framework for integrated exploration and overview of various databases in the life science field, enabling users to extract necessary information from a vast amount of information by flexibly narrowing down information using various attributes [6]. In the “Condition Builder” in the upper right corner of the TogoDX screen, under the Filtering setting, select Human phenotype ontology in the target dataset. Then, in the main screen of TogoDX, under the selection of Phenotypic abnormalities, the list of terms in each category was obtained by selecting each one of the 23 categories directly below it and downloading the terms inside as a tsv file. The design of TogoDX allows only last leaf nodes in a selected category of HPO to be displayed and output in tsv file.

### Categorization of terms in HPO with LLM or by manual curation

ChatGPT(GPT-4 turbo) was employed to extract all HPO terms for each of the 23 HPO subclasses (defined as categories) directly under the HPO "Phenotypic abnormality". We created GPTs to deal with the terms in the HPO and uploaded an Excel file of the list of terms of HPO phenotypic abnormality.

The instructions of GTPs were as follows, “The HPO Categorizer processes terms from an uploaded file containing a large category of HPO (Human Phenotype Ontology) terms. Each term is categorized into four main categories: Disease Names, Conditions, Test Data, and Symptoms and Findings. Multiple tags can be assigned to a single term. The categorizer also uses web search to gather additional information when needed. After categorization, the terms are output as an Excel file. The Excel file will have columns for the term ID and label on the left, followed by four columns for the categories, where each cell is marked with a “ + ” or “ − ” to indicate the presence or absence of the category tag for each term.” The “Conversation starters” were as follows: “Categorize this term from the uploaded file.”,” Search for additional information on this HPO term.”,” Generate an Excel sheet for these HPO terms.” and “Tag this term using the 4 HPO categories.”.

The following prompts were used to categorize terms in each category of HPO.

“Please categorize the terms in the uploaded files according to the following categories: “Disease Names”, “Conditions”, “Test Data”, and “Symptoms and Findings”, using the uploaded file and website as a reference”. All the outputs were evaluated from the clinician’s perspectives.

We also manually categorized and tagged the terms in each of these category listings into four major attributes: “Disease Names,” “Conditions,” “Test Data”, and “Symptoms and Findings”. These procedures were evaluated and confirmed from a clinician’s perspectives.

## Results

### GPTs was not sufficient to extract the terms in each category of HPO phenotypic abnormality

We attempted to generate the list of terms in each category of HPO with GPTs uploaded with Excel files corresponding to HPO. However, we could not extract the terms and could not generate the list of terms in each category, even after applying both 2 files of HPO and with the effort to improve the prompts. Therefore, we explored the other possibility where we can get the list of HPOs in each category and employ TogoDX. We successfully generated the list of HPO terms in each category of Phenotypic abnormality, and the number of terms in each category ranged from 4 to 2743 and the average number of terms in one category was 676.8 (Table 1).

**Table 1:** Number of HPO in each category (leaf nodes)

**categories within Phenotypic Abnormality**	**N**
**Abnormality of the genitourinary system**	**1039**
**Abnormal cellular phenotyp**	**191**
**Abnormality of blood and blood-forming tissues**	**570**
**Abnormality of head and neck**	**961**
**Abnormality of limbs**	**1688**
**Abnormality of metabolism/homeostasis**	**1219**
**Abnormality of prenatal development or birth**	**192**
**Abnormality of the breast**	**24**
**Abnormality of the cardiovascular system**	**957**
**Abnormality of the digestive system**	**496**
**Abnormality of the ear**	**213**
**Abnormality of the endocrine system**	**273**
**Abnormality of the eye**	**777**
**Abnormality of the immune system**	**871**
**Abnormality of the integument**	**713**
**Abnormality of the muscleskeletal system**	**2743**
**Abnormality of the nervous system**	**1586**
**Abnormality of the respiratory system**	**445**
**Abnormality of the thoracic cavity**	**4**
**Abnormality of the voice**	**21**
**Constitutional symptom**	**81**
**Growth abnormality**	**68**
**Neoplasm**	**435**

### GPTs was not sufficient to categorize the terms in each category of HPO even with web search

A manual search for terms within each category revealed that the HPO contains a mix of terms with four major attributes: (1) disease name, (2) condition, (3) test data, and (4) symptoms and findings (which can be revealed by physical examination). Of these, the “Test Data” in particular could be classified into findings (detectable with tools and specialized equipment), biochemical tests of body fluids, physiological tests, imaging tests, and pathological tests, which could be further subdivided into more detailed and specialized tests. We also found that a term can act as different attributes depending on the context in which it is used and that a term can have multiple attributes. Therefore, in order to capture the characteristics of each category, we explored the proportion of attributes latent in each category for this term. We attempted to categorize the terms in each category of HPO with GPTs uploaded with corresponding Excel files (available on GitHub). As an example, the analysis of “Abnormality of head or neck.xlsx” revealed that out of total 961 terms, only 14 were categorized as symptoms and findings with GPTs (Categorized_Abnormality_of_head_or_neck.xlsx, available at GitHub). Manual curation of this data by clinicians resulted in 895 of the 961 terms (93.1%), being classified as Symptoms and findings. The results were clearly divergent from a clinician’s perspective as end-users, and it is clear that this approach is inadequate to categorize into four major attributes.

### The characteristics of terms in each category of HPO were diverse

Given the use of LLM as tested above, we changed our strategy to categorize the terms into four major attributes with manual curation. The curation of 16 categories revealed a wide distribution of symptoms and findings, ranging from 0 to 93.1% per category (Table 2). These suggested that each category had significant characteristics, and we looked at the data in more detail. For example, symptoms and findings appeared very frequently in the dermatology and voice categories. This suggests that there are many abnormalities that can be detected by physical examination. In addition, endoscopic findings were very common in the gastrointestinal category, and radiographic findings were very common in the respiratory and musculoskeletal categories. In addition, all terms in neoplasm were names of cancer.

**Table 2:** Number of Symptoms/Findings (which can be revealed by physical examination)

	**Total**	**Symptoms/Findings**	**%**
**Abnormal cellular phenotyp**	**191**	**0**	**0.0 **
**Abnormality of blood and blood-forming tissues**	**570**	**26**	**4.6 **
**Abnormality of head and neck**	**961**	**895**	**93.1 **
**Abnormality of prenatal development or birth**	**192**	**33**	**17.2 **
**Abnormality of the breast**	**24**	**19**	**79.2 **
**Abnormality of the digestive system**	**496**	**92**	**18.5 **
**Abnormality of the ear**	**213**	**154**	**72.3 **
**Abnormality of the endocrine system**	**273**	**5**	**1.8 **
**Abnormality of the immune system**	**871**	**31**	**3.6 **
**Abnormality of the integument**	**713**	**603**	**84.6 **
**Abnormality of the respiratory system**	**445**	**67**	**15.1 **
**Abnormality of the thoracic cavity**	**4**	**0**	**0.0 **
**Abnormality of the voice**	**21**	**16**	**76.2 **
**Constitutional symptom**	**81**	**77**	**95.1 **
**Growth abnormality**	**68**	**63**	**92.6 **
**Neoplasm**	**435**	**0**	**0.0 **

## Discussion

While LLM has made it possible to extract symptoms and findings from clinical texts, it has been difficult to annotate them with HPO. Then, we hypothesized that one possible reason for this would be the lack of appropriate words for HPO, and during the BLAH8, we attempted to capture the whole picture of HPO in a way that would be easy to understand for the end-user such as the clinician. We put 2 steps (1) extract all HPO terms for each of the 23 HPO subclasses directly under HPO "Phenotypic abnormality" and (2) categorize terms into 4 major attributes “Disease Names”, “Conditions”, “Test Data”, and “Symptoms and Findings”. For these 2 tasks, we employed GPTs (ChatGPT 4.0) with corresponding files and web search; however, both of which yielded poor quality results. The ability to extract symptoms and findings from clinical text using LLMs may be due to the presence of context. On the other hand, the training data for LLM may not be enough to examining the HPO, a highly specialized ontology, and may not have adequately addressed these two tasks.

In order to produce highly specialized, detailed, and precise data, it should be important to augment the ontology. Such data is also important for improving the quality of LLMs and creating function-specific LLMs. However, in order to have experts work on ontology, which is difficult to read because of its tree structure, we need to ask what is missing and what is not missing with respect to the ontology. We believe that it will be important to visualize this point in an easy-to-understand manner, especially for clinicians, the end-users of the HPO, and experts in medical terminology.

HPO is organized in a Directed Acyclic Graph (DAG) that counts multiple parent concepts as separate ones (https://www.informatics.jax.org/userhelp/VOCAB_hpo_browser_help.shtml). On the other hand, TogoDX displays and extracts only the leaf nodes of the selected category, so it does not purely mean that all terms within each category have been extracted and the number of terms extracted is reduced. However, the leaf nodes are endophenotypes that reflect the characteristics of the category to some extent. As shown in Table 2, even if we limit the terms such as symptoms and findings, we find diverse proportions among the categories within HPO. Such information helps the end-user clinician, who is not usually exposed to ontologies but understands the terminology well enough to use it on a daily basis, to grasp the overall picture of the HPO.

The tagging of the four major attributes that was done this time needs to be re-examined by more specialized medical professionals. However, we believe that even in a somewhat rough state, as in this case, the creation of a draft of the tagged HPO list will make it easier to grasp the overall picture and provide a situation in which medical professionals can easily work on the HPO list. In addition, through these efforts, it will be possible to determine the extent to which the ontology covers the medical terminology end-user. This will also help the end-users of medical terminology, i.e., clinicians, to better understand how far the ontology covers, which will in turn enhance the explanatory power of artificial intelligence, including LLM, that uses the ontology.

Through BLAH8, we were able to attempt to build a platform for augmenting the fundamental knowledge of ontologies. In addition to this, there are still many unexplored aspects of how to annotate with such a newly tagged ontology and how to utilize the corpus after annotation, and we will continue our research on how to further utilize ontologies and corpora in the future.

## Data Availability

The curated Excel files of HPO for each category are available on GitHub (https://github.com/domy1980/Examining-HPO-by-organ-and-system-to-facilitate-practical-use-by-clinicians).

## References

[1] Gargano MA, Matentzoglu N, Coleman B, Addo-Lartey EB, Anagnostopoulos AV, Anderton J, et al. The Human Phenotype Ontology in 2024: phenotypes around the world. Nucleic Acids Res. 2024;52(D1):D1333–46. 10.1093/nar/gkad1005. 37953324 10.1093/nar/gkad1005 PMC10767975

[2] Lima-López S, et al. Overview of SympTEMIST at BioCreative VIII: corpus, guidelines and evaluation of systems for the detection and normalization of symptoms, signs and findings from text. Proceedings of the BioCreative VIII Challenge and Workshop: Curation and Evaluation in the era of Generative Models. 2023.

[3] Ling L, Shankai Y, Po-Ting L, Daniel V, Andrew O, Sandhya X, Rajarshi G, Morgan S, Peter NR, Zhiyong L. PhenoTagger: a hybrid method for phenotype concept recognition using human phenotype ontology. Bioinform. 2021;37(13):1884–90. 10.1093/bioinformatics/btab019. 10.1093/bioinformatics/btab019 PMC11025364 33471061

[4] Eisuke D, Ali HB. Visualizing the phenotype diversity: a case study of Alexander disease. Genomics Inform. 2021;19(3):e28 PubMed PMID: 34638175; PubMed Central PMCID: PMC8510876. 34638175 10.5808/gi.21016 PMC8510876

[5] Dohi E, Kushida T, Yamagata Y, Takatsuki T, Shin J, Masuya H, et al. BioHackJP 2023 Report R1: Improving phenotype ontology interoperability. BioHackrXiv Preprints. 10.37044/osf.io/d27fw.

[6] TogoDX. Available from: https://togodx.dbcls.jp/human/. Accessed Jan 2024.

